# No Confederates Need Apply

**Published:** 1893-11

**Authors:** 


### NO CONFEDERATE NEED APPLY.

We read with surprise and an indignation too profound to be expressed in words—a late Washington dispatch that Mr. Lochren, Mr. Cleveland's new Commissioner of Pensions, has ruled that "no one who served in the Confederate service, should be eligible to a position on the Board of Medical Examiners for Pensions!" And this, the dispatch stated, has Mr. Cleveland's approval!

Confederate major generals and Confederate brigadier generals are in the congress of the United States; and a very conspicuous Confederate, L. Q. C. Lamar, graced the Supreme Bench of the United States court; but a *doctor* who had the misfortune to be born in the South, and whose sympathies and services were of *course* given to his State, is disqualified by that mere fact, whatever may be his ability, experience or superior fitness for the position, for the examination of applicants for pensions. It is monstrous; it is outrageous; an indignity gratuitously offered to the medical profession of the South by this appointee of Mr. Clevleand's, and Mr. Cleveland endorses it. Whose votes elected Mr. Cleveland,—if not that of the South?

Every old resident physician in the South, perhaps, who is now over fifty or fifty-five years of age, served in the Confederate army; most of them as medical officers. Their experience with wounded and disabled soldiers; their familiarity with influences on health of the camp, the march, the army regimen, the defective personal hygiene of the soldier, etc., renders them peculiarly fitted to estimate the extent of disability, and the causes and their relation, in those who apply for pensions; and their acquaintance with the deceptions practiced by soldiers in order to shirk duty, the tricks of the *malingerers*, renders the ex-Confederate surgeons especially capable of detecting imposition; and if the millions now carried on the pension rolls of the United States, were carefully re-examined by a board of ex-Confederate surgeons, doubtless thousands would be found fraudulent and unworthy of the pension they are now drawing; and the $185,-

000,000 annually paid as pensions to the ex-Federal soldiers would be materially reduced. Is that what Mr. Lochren is afraid of?

The physicians of the South should not only remember this outrage but should protest against it by every means in their power.

